# Effect of Microwave Vacuum Drying on the Drying Characteristics, Color, Microstructure, and Antioxidant Activity of Green Coffee Beans

**DOI:** 10.3390/molecules23051146

**Published:** 2018-05-11

**Authors:** Wenjiang Dong, Ke Cheng, Rongsuo Hu, Zhong Chu, Jianping Zhao, Yuzhou Long

**Affiliations:** 1 Spice and Beverage Research Institute, Chinese Academy of Tropical Agricultural Sciences, Wanning 571533, China; dongwenjiang.123@163.com (W.D.); chengke005@126.com (K.C.); hnhrs@126.com (R.H.); cz809@163.com (Z.C.); 2 College of Food Science and Technology, Huazhong Agricultural University, Wuhan 430070, China; 3 Tropical Crops Genetic Resources Institute, Chinese Academy of Tropical Agricultural Sciences, Danzhou 571700, China; zjp-68068@163.com

**Keywords:** green coffee bean, microwave vacuum drying, mathematical modeling, kinetics, antioxidant activity

## Abstract

The aim of this study is to investigate the effect of microwave vacuum drying (MVD) on the drying characteristics and quality attributes of green coffee beans. We specifically focused on the effective moisture diffusion coefficient (*D_eff_*), surface temperature, glass transition temperature (*T_g_*), water state, and microstructure. The kinetics of color changes during drying, total phenolic content (TPC), and antioxidant activity (DPPH, FRAP, and ABTS) were also characterized. Microwave power during MVD affected the porosity of coffee beans, their color, TPC, and antioxidant activity. The Allometric 1 model was the most suitable for simulating surface temperature rise kinetics. Thermal processing of green coffee beans resulted in increased b*, L*, Δ*E*, and TPC values, and greater antioxidant capacity. These findings may provide a theoretical reference for the technical improvement, mechanisms of flavor compound formation, and quality control of dried green coffee beans.

## 1. Introduction

Coffee is one of the most popular beverages consumed by people around the world. Furthermore, it is the most important agricultural product whose production brings considerable economic benefits to certain developing tropical countries [1]. The two main commercial species of coffee are *Coffea canephora* and *Coffea arabica*, which account for 40% and 60%, respectively, of commercial coffee. The Hainan province is the main *C. canephora* cultivation area in China [2]. Once harvested from the coffee trees, coffee cherries rot rapidly unless they are quickly processed. Dehydration is probably the oldest method for the prevention of food rot; it is used to preserve coffee cherries, also reducing their size, and cost of storage and transportation [3]. The wet and dry methods, the two types of coffee dehydration, determine the characteristic coffee flavor. During wet processing, the flesh of the coffee cherry is removed by mechanical de-pulping, which is followed by a fermentative degradation of the remaining mucilage before coffee beans are dried. In contrast, during dry processing, the coffee cherries are dried directly before they are hulled to obtained coffee beans. However, it has been shown that various metabolic activities present in green coffee beans are preserved in the course of wet processing [4].

Solar drying is a dehydration method that is widely used in post-harvesting processing of agricultural products. However, it is a lengthy process, and solar-dried coffee beans undergo some undesirable physical and chemical changes. In recent years, microwave vacuum drying (MVD) has been widely used for drying of agricultural products, such as lotus seeds and blueberries [5,6]. The microwave generates a specific quantity of energy, conveniently shortening the drying time. In addition, the boiling point of water is lowered under vacuum, leading to a higher temperature inside the dried particles than on the surface of the product. This phenomenon increases the partial pressure that drives the evaporating water to the outer layer. Thus, MVD can reduce the dehydration time and prevent deterioration of the quality of perishable materials [7].

Currently, the drying processes are usually described using phenomenological or empirical models. Kinetic modeling not only describes the drying kinetics of materials but also informs the design and optimization of the technological parameters of dryers. Two important aspects of drying simulation models should be considered; these are: estimation of the drying rates for thin-layer drying and the effective moisture diffusion factor of the material. These two aspects also constitute the foundation of moisture transfer analysis [8]. Surface temperature (ST) is an important index for controlling the quality of materials. Temperature rise kinetics is a helpful tool for predicting the surface temperature of coffee beans during MVD. Infrared thermal imaging is used to detect the hot spots in materials during drying, providing an immediate visual map of heat distribution [9]. If the microwave power during MVD is not controlled well, the treated material may easily overheat. Thus, it is important to monitor the heat distribution of the treated material during MVD. 

Color is one of the parameters used for process control during MVD because non-enzymatic browning is positively correlated with the drying temperature [10]. Monitoring of color changes of coffee beans during MVD is necessary for controlling and optimizing the MVD process. In addition to color, structure is another important control parameter of coffee bean drying. The cellular structure of foods dried by MVD is porous because of the vacuum and internal vapor pressure. Such porous structure is often associated with a more complete rehydration and greater water retention than samples dried by vacuum drying (VD) [11]. The water state of coffee beans during MVD is also an important factor determining the quality of dried coffee beans. Nuclear magnetic resonance (NMR) and magnetic resonance imaging (MRI) are used to monitor water mobility and the distribution of the water state in materials during MVD. An additional parameter, glass transition temperature (*T_g_*), affects the stability of green coffee beans during storage and their processing characteristics. This is because a food system is relatively stable, with a long shelf time, at temperatures below *T_g_*. Evaluation of the *T_g_* of green coffee beans during MVD is, therefore, a good approach for improving the preservation of the beans. To the best of our knowledge, no studies on the microstructure, *T_g_*, and water state of green coffee beans during MVD have been reported to date.

Utilization of green coffee beans recently gained considerable attention in the nutraceutical and pharmaceutical industries because of their high antioxidant content and radical scavenging activities [12]. Phenolic compounds are a major resource of antioxidant activity, and also have some other beneficial physicochemical and biological properties [13]. Green coffee beans exhibit a high level of antioxidant activity, which may be associated with their total phenolic content (TPC) [14]. The antioxidant capacity and TPC were evaluated in dried coffee beans; however, no data have been published on the kinetics of changes in TPC and antioxidant compounds in coffee beans during thermal drying. Some authors have reported an increase in the antioxidant activity of fruits and vegetables during thermal processing [15], while others showed the opposite [13,16].

In the present study, we evaluated the properties of wet green coffee beans (obtained by the wet processing method) during MVD. The aims of the present study were to (1) determine the drying and surface temperature rise kinetics of green coffee beans during MVD; (2) evaluate the water state, glass transition temperature, and microstructure of coffee beans during MVD by infrared thermal imaging and MRI; (3) determine the changes of the physical and chemical characteristics of green coffee beans during different microwave power treatments, including color, TPC, and antioxidant capacity. The findings may provide a theoretical basis for explaining the changes in flavor compounds during coffee bean processing; for informing technical improvement of coffee bean processing; and for quality control of dried green coffee beans.

## 2. Results and Discussion

### 2.1. Drying Characteristics and D_eff_

**Figure 1:**
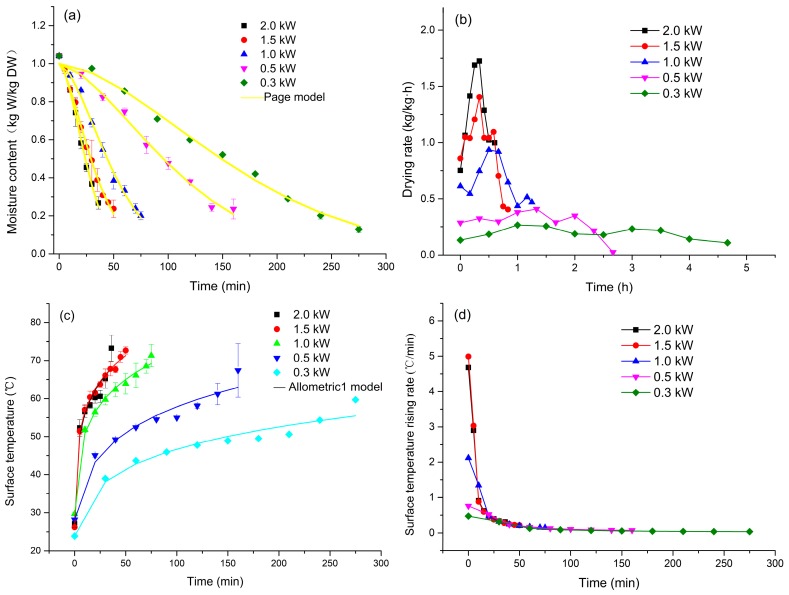
Effect of microwave power setting on drying and surface temperature rise curves of green coffee beans during MVD at −0.085 mPa: (**a**) Moisture ratio vs. drying time; (**b**) drying rate vs. drying time; (**c**) surface temperature vs. drying time; and (**d**) surface temperature rising rate vs. drying time.

The experimental drying curves of green coffee beans were obtained using MVD at five microwave power settings: 0.3 kW, 0.5 kW, 1.0 kW, 1.5 kW, and 2.0 kW, at the absolute pressure of −0.085 mPa (Figure 1a). With an increasing microwave power, the Moisture Ratio (MR) for MVD of the same duration decreased and the total drying time was reduced. The final moisture content of green coffee beans treated at each microwave power setting reached 0.11 (g/g db). The drying time required at the 0.3 kW treatment was 280 min, and it was reduced with increasing power. Moreover, the drying period showed a linear trend when the microwave power was set to 0.3 kW or 0.5 kW. In contrast, two drying periods were observed for the other treatments: initially, the moisture content decreased rapidly; it then slowly decreased until equilibrium was reached. Zhao et al. [5] reported similar observations for lotus seeds when the drying densities were 10.0 W/g, 15.0 W/g, and 20.0 W/g.

Figure 1b presents the differences in the drying rates of green coffee beans at five microwave power settings. The increasing drying rate could be attributed to high microwave energy absorption by a substantial number of dipole molecules, if present, while the decreased drying rate may have been associated with the internal resistance to both heat and mass transfer [17]. Moreover, the drying rate accelerated more rapidly at higher microwave power settings, with the highest value of 1.73 kg water/kg DW/h at 2.0 kW. In contrast, the drying rate appeared to change negligibly at 0.5 kW and 0.3 kW. The tendency of the drying rate to become uniform may have been associated with the low microwave power and high material weight (2.0 kg). In addition, the effective moisture diffusivity (*D_eff_*) (Table 1) increased as the microwave power increased. This was because the increasing heating energy accelerates the kinetic energy of water molecules, increasing moisture diffusivity [5]. *D_eff_* was 1.13 cm^2^/min at 0.3 kW, and it increased by 50.44%, 234.51%, 392.92%, and 485.84% at microwave power settings of 0.5 kW, 1.0 kW, 1.5 kW, and 2.0 kW, respectively. This phenomenon was accounted for an increase of microwave power in material, which may cause moisture inside spread fast to the surface, thus the *D_eff_* increased.

### 2.2. Surface Temperature Rise Characteristics

The surface temperature (ST) rise curves of green coffee beans dried at the different microwave power settings are shown in Figure 1c. The ST increased with increasing microwave power, with the curves exhibiting a unique convex shape, namely, an initial quick increase of the rising rate, followed by a slow increase of the rate. The maximum ST was 73.23 °C, at microwave power of 2.0 kW. ST was reduced to 72.65 °C, 71.31 °C, 67.41 °C, and 59.70 °C at microwave power settings of 1.5 kW, 1.0 kW, 0.5 kW, and 0.3 kW, respectively.

Figure 1d shows the rate of ST rise of green coffee beans at the different microwave power settings vs. the drying time. The rate of ST rise decreased with increasing time, although a relatively rapidly increasing rate was observed at beginning. The initial rate of ST rise at microwave power of 2.0 kW and 1.5 kW was similar. It decreased to 56.0%, 84.0%, and 90.0% at the microwave power of 1.0 kW, 0.5 kW, and 0.3 kW. The drying rates at 0.5 kW and 0.3 kW were uniform, in accordance with the rates of ST rise. Therefore, the ST was an important parameter that impacted the drying characteristics of green coffee beans. In addition, the almost uniform rate of ST rise could be attributed to the lighter material weight (2.0 kg). The reports on surface temperature rise kinetics are not numerous, although Jiang et al. [18] reported on the surface and interior temperature rise kinetics of banana chips during microwave freeze-drying. However, because a detailed surface temperature of a material at each time point of drying has not yet been reported, Allometric 1 model was used to model the dynamic changes of surface temperature of coffee beans during MVD.

### 2.3. Drying Kinetic and Surface Temperature Rise Kinetics

Experimental drying data were analyzed statistically to obtain the most suitable drying model. The results are summarized in Supplemental Table S1. The best model for describing the drying characteristics of coffee beans was selected based on high *R*^2^, and low *χ*^2^ and *RMSE* values. As shown in Supplemental Table S1, 10 models were characterized by high *R*^2^, and low *χ*^2^ and *RMSE* values were close to 0.90, 0.05, and 0.01, respectively. Among the models examined, the Page model was the best one for describing MVD at 2.0 kW, 1.0 kW, and 0.5 kW. However, the approximation of diffusion model and logarithmic model were the best ones for MVD at 1.5 kW and 0.3 kW, respectively. Because in the Page model, the *R*^2^ value was higher, and *χ*^2^ and *RMSE* values were lower than in the remaining nine models, the Page model was chosen for fitting the five curves from Figure 1a. In contrast, Chen et al. [19] reported that the logarithmic model and the two-term model were the best models for describing the drying process of jujube slices during hot-air, and short- and medium-wave infrared radiation drying, respectively. The application of Allometric 1 model to ST rise of green coffee beans enabled dynamic monitoring of the temperature change of green coffee beans during MVD (Table 1). For the kinetic parameters *k* and *b*, the value of *k* increased with the microwave power, while the change of *b* was negligible. The Allometric 1 model fitted the drying data at the microwave power setting of 1.5 kW better than drying data at any other microwave power setting.

### 2.4. Differential Scanning Calorimetry Analysis

Differential Scanning Calorimetry (DSC) was employed to characterize the chemical and physical changes of coffee beans during drying. Tremendous efforts have been made by researchers to study the heat interactions and thermal stability of green coffee beans over the past few decades [20]. However, the effect of MVD on the thermodynamic properties of green coffee beans was not examined in detail. In the present study, the heat flow differences between the sample and the reference (an empty aluminum pan) were recorded as a function of temperature, which increased at a constant rate for both the sample and the reference. The heat flow was equivalent to enthalpy because the pressure was kept constant [21]. Changes in the thermal behavior of dried coffee beans after MVD at different microwave power settings between 20 °C and 250 °C, and at a heating rate of 10 °C/min under constant nitrogen atmosphere, are shown in Supplemental Figure S1. The thermograms of the five different dried coffee bean samples revealed an exothermic event, which occurred because of the vaporization of water and crystalline nature of the sample. The enthalpy values associated with this event were 136.8 J/g, 148.1 J/g, 160.9 J/g, 179.1 J/g, and 152.8 J/g, at 2.0 kW, 1.5 kW, 1.0 kW, 0.5 kW, and 0.3 kW, respectively. Further, the peak temperatures of the exothermic events were 128.87 °C, 130.53 °C, 127.74 °C, 128.81 °C, and 126.72 °C, respectively, at decreasing microwave power settings. Analysis of the DSC thermograms revealed inflexions associated with glass transition, at around 59.88 °C, 60.37 °C, 60.85 °C, 61.76 °C, and 61.45 °C, respectively, at decreasing microwave power settings. Nevertheless, the differences between these five dried coffee bean preparations were not significant.

### 2.5. Water State Determination by Time-Domain Nuclear Magnetic Resonance

During time-domain nuclear magnetic resonance (TD-NMR) spectrometry, *T*_2_ weighted relaxation curves were obtained for a detailed overview of the distribution of water inside the cellular structures of green coffee beans as a function of microwave power. The water in food can be divided into the following fractions: bound water, cytoplasmic bulk water, and free water. These fractions can be differentiated by NMR based on the relaxation time, which is dependent on the strength of their binding to food. The relaxation time of three types of signal peaks, from short to long, represents bound water (0.01 ms < *T*_21_ < 10.0 ms), cytoplasmic bulk water (10.0 ms < *T*_22_ < 70.0 ms), and free water (*T*_23_ > 70.0 ms). *T*_22_ represented the main population group of water present in green coffee beans, and the initial distributions of *T*_21_ and *T*_23_ were almost equal. This is commonly observed in fruits and vegetables [22].

**Figure 2:**
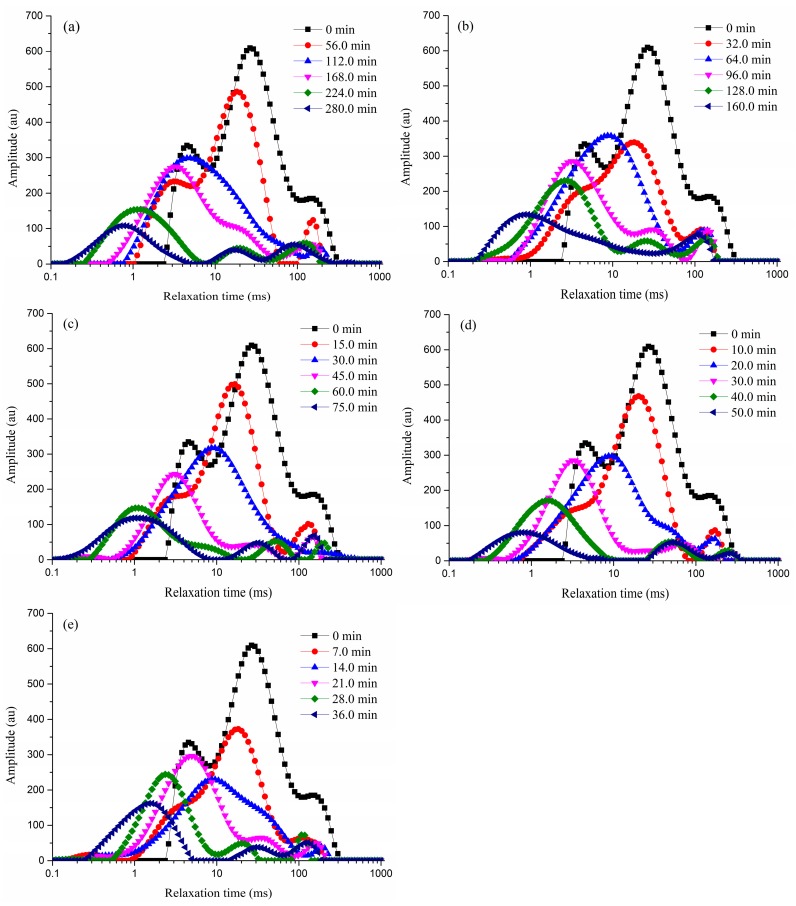
Distribution of T2 relaxation times of samples that underwent MVD at different microwave power settings: (**a**) 0.3 kW; (**b**) 0.5 kW; (**c**) 1.0 kW; (**d**) 1.5 kW; and (**e**) 2.0 kW.

Figure 2a–e shows the distribution of *T*_2_ relaxation times of samples treated by MVD at the different microwave power settings. As the drying time increased, the peak positions of samples shifted to the left of the signal intensity-*T*_2_ curve in all samples. This indicated that the five MVD treatments resulted in reduced water fluidity during drying. These observations were in agreement with the reports of Jiang et al. [18] and Zhao et al. [5]. The changes of peak area ratio of bound water (A_21_), cytoplasmic bulk water (A_22_), and free water (A_23_) in coffee beans are presented in Supplemental Table S2. The A_21_ values first decreased, which may have been caused by the shift of free water to cytoplasmic bulk water, following which the percentage of bound water decreased. The latter may be explained by the increase in the concentration of carbohydrates and the degradation of nutritional components in the cytoplasm [23]. However, the A_21_ values then increased, which may have been caused by the shift of the cytoplasmic bulk water to bound water. This may explain the variation of the A_22_ values. Further, the A_23_ values remained almost unchanged. The changes of A_21_ and A_22_ values were more pronounced at higher microwave power settings; this phenomenon might be associated with excessive and rapid sample heating, resulting in cell membrane disruption.

### 2.6. NMR Imaging

Representative images of green coffee beans treated by MVD at 1.0 kW are shown in Supplemental Figure S2a–f. The moisture content of green coffee beans is high (approximately 51.0%, *w*/*v*) and, hence, the beans are very bright in the image. The brightness decreased as the drying time increased. During imaging, sample area becomes darker if the sample contains a high concentration of ions or a contrast agent, etc., which enhances relaxation [24]. The dehydration of green coffee beans was visualized in the successive images, and the coffee bean color was similar to the background color after the drying was completed. Furthermore, the image of the green coffee beans was more colorful than that of dried coffee beans, indicating that water content and water state were reduced during MVD. To the best of our knowledge, the present study is the first to use NMR imaging to analyse the drying process of coffee beans.

### 2.7. Color Analysis

**Figure 3:**
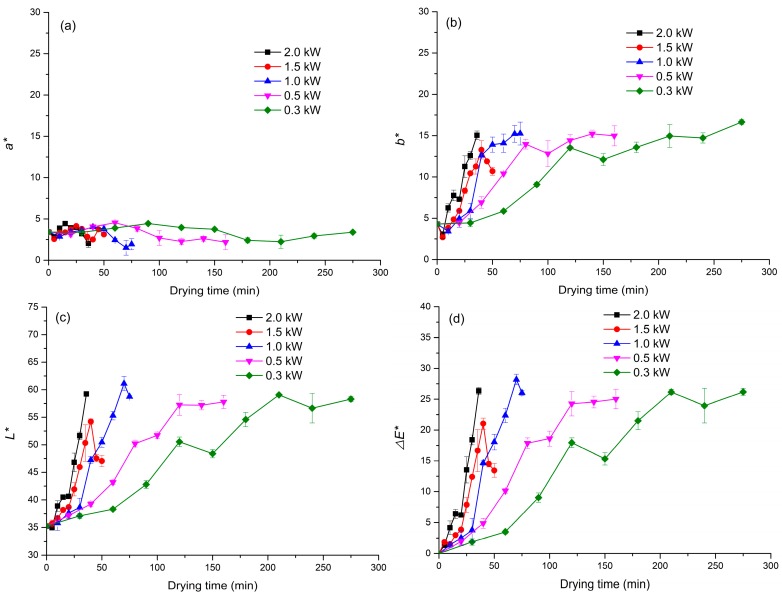
Kinetics of the color parameter changes of coffee beans during MVD: (**a**) a*; (**b**) b*; (**c**) L*; and (**d**) Δ*E*.

Color is one of the most important factors influencing the sensory attributes of food products, and therefore plays an important role in consumer evaluation of food quality. Generally, the b* and L* values increased with the increasing duration of drying of green coffee beans. In contrast, a* remained nearly constant, and there were obvious distinct of the Δ*E* values during MVD of green coffee beans. L* values of green coffee beans increased with the drying process (Figure 3a–d). This indicated that the color of dried coffee beans became lighter than that of green coffee beans, which was consistent with other reports [25]. Similarly, b* values of dried coffee beans were higher than those of fresh beans, indicating that the yellowness of green coffee beans increased after drying. However, a* values of green coffee beans and dried coffee beans were almost identical and between 0–5, indicating that the redness of green and dried coffee beans was shallow. The overall color change of the dried product is important, as the human eye is able to discriminate samples based on color [26]. The overall color difference (Δ*E*) of dried coffee beans was higher than that of green coffee beans; Δ*E* of green coffee beans during drying changed mainly because of the increase in L* and b*. Further, the values of L*, b*, and Δ*E* increased more rapidly at higher microwave power settings. In contrast, Zhao et al. [5] reported that the values of L* and b* of lotus seeds decreased, while the value of a* increased with increasing drying time.

### 2.8. Infrared Thermal Imaging

Infrared thermal imaging is a non-destructive technique, which is widely applied in the food industry. Infrared thermal imaging transforms the thermal energy, radiating from objects in the infrared band of the electromagnetic spectrum, into a visible image; each energy level is represented by a color or on a gray scale [9]. Infrared thermal imager is basically a camera with an infrared detector, and heat distribution on the surface of a material is observed as a difference in color. A typical thermography analysis of green coffee beans dried at a microwave power setting of 1.0 kW is shown in Supplemental Figure S3a–h. The surface temperature of green coffee beans was in the range of 21.9–27.6 °C (temperature difference of 5.7 °C). As the drying process continued, the temperature differences of dried coffee beans increased with drying time. The inhomogeneity of MVD-treated samples was evident after more after 50 min of drying, and became more severe as the drying continued. Therefore, to minimize the inhomogeneity of MVD processing, it MVD may be replaced by a different uniform drying process after 50 min.

### 2.9. Scanning Electron Microscope Analysis

**Figure 4:**
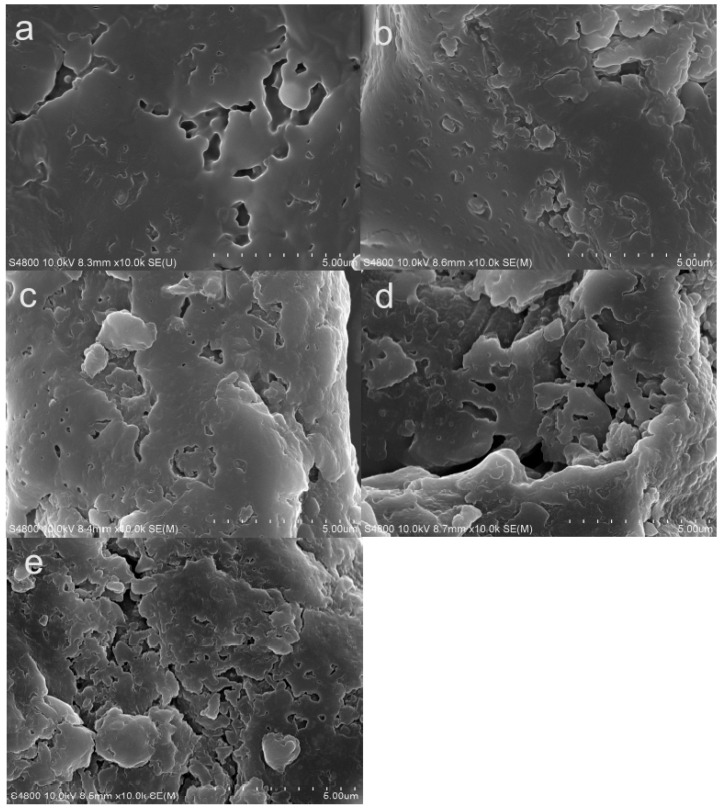
Microstructure of coffee samples dried at different microwave power settings: (**a**) 0.3 kW; (**b**) 0.5 kW; (**c**) 1.0 kW; (**d**) 1.5 kW; and (**e**) 2.0 kW.

SEM images of the microstructure of green coffee beans dried at different microwave power settings are shown in Figure 4a–e. The microwave drying power setting had a pronounced effect on the microstructure of dried coffee beans. In general, MVD first resulted in an initial moisture loss of green coffee beans, and pore structure was apparent. As shown in Figure 4e, the pores in coffee powder were larger and the fragmentation of structure was more apparent at higher microwave power. However, the pores became less numerous and smaller with a decreasing microwave power; the same was noted for the extent of structure fragmentation. Tian et al. [11] reported similar observations for shiitake mushrooms during MVD. This may have been because the evaporation rate of water was faster and the cell structure was more disrupted during processing at a higher microwave power setting, resulting in loose pore structure. In contrast, the surface temperature rose slowly and, hence, the water evaporation rate was slower at lower microwave power, resulting in the preservation of most cellular structures and smaller pores.

### 2.10. Total Phenolic Content Analysis

**Figure 5:**
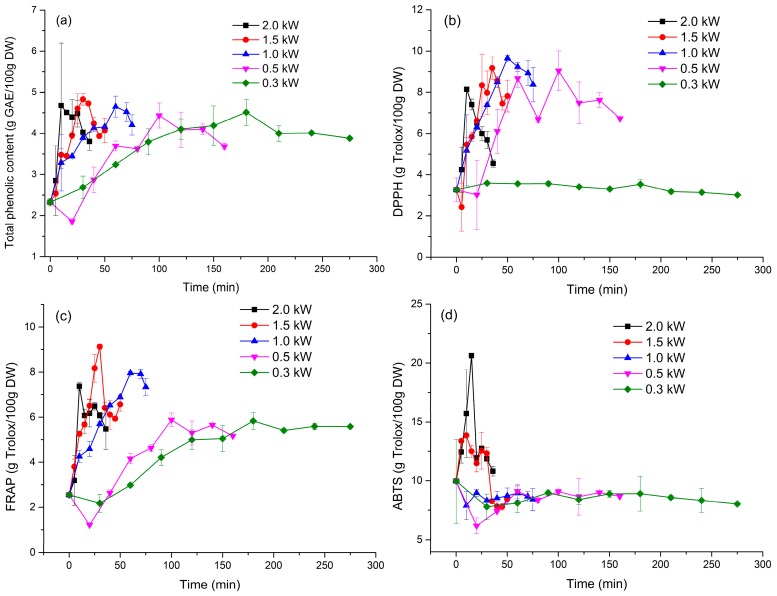
Kinetics of total phenolic content and antioxidant activity of coffee beans during MVD: (**a**) TPC; (**b**) DPPH; (**c**) FRAP; and (**d**) ABTS.

MVD resulted in a significant increase of TPC, as green coffee beans contained 2.33 ± 0.09 g GAE/100 g DW, while dried coffee beans contained between 2.54 ± 0.08 GAE/100 g DW (9.0% increase of the initial TPC) and 4.83 ± 0.14 g (107.0% increase of the initial TPC) (Figure 5a). The TPC showed a tendency to first increase and then decrease as the drying continued. In addition, the rate of increase was higher at higher microwave power settings. The highest TPC content during drying stage was observed at 10.0 min, 30.0 min, 60.0 min, 100.0 min, and 180.0 min at microwave power setting of 2.0 kW, 1.5 kW, 1.0 kW, 0.5 kW, and 0.3 kW, respectively. Zheng et al. [27] observed the opposite, i.e., TPC decrease during the drying of loquat flower tea. This may be because tea polyphenols decompose more easily during drying. The time when the highest TPC content was first observed coincided with the time when the temperature differences of dried coffee beans determined by infrared thermal imaging were largest.

### 2.11. Antioxidant Activity Determinations

Coffee beans were rich antioxidant sources, as determined by the DPPH, FRAP, and ABTS analyses. The DPPH free radical assay is widely accepted as a tool for estimating the free radical-scavenging activity of antioxidants because of the stability of the radical. The DPPH values remained almost unchanged during drying at a microwave power of 0.3 kW. However, they tended to increase when the drying was conducted at higher microwave power settings (Figure 5b). The highest DPPH values were observed after 10.0 min, 35.0 min, 50.0 min, and 100.0 min at 2.0 kW, 1.5 kW, 1.0 kW, and 0.5 kW, respectively. The values (g Trolox/100 g DW) were 8.14 ± 0.02, 9.17 ± 0.54, 9.65 ± 0.13, and 9.04 ± 0.97 at 2.0 kW (after 10 min), 1.5 kW (35 min), 1.0 kW (50.0 min), and 0.5 kW (100.0 min), respectively. The DPPH scavenging ability was significantly correlated with TPC (*R*^2^ = 0.660, *p* < 0.01) and FRAP (*R*^2^ = 0.706, *p* < 0.01), but not with ABTS (*R*^2^ = 0.070, *p* < 0.01) (Supplemental Table S3). Indeed, strong correlation between TPC and antioxidant activity was also reported in other studies [13,28].

As shown in Figure 5c, the FRAP values also tended to increase during drying. The green coffee beans contained 2.55 ± 0.02 g Trolox/100 g DW. The highest FRAP values during drying were after 10.0 min, 30.0 min, 60.0 min, 100.0 min, and 180.0 min with microwave power settings of 2.0 kW, 1.5 kW, 1.0 kW, 0.5 kW, and 0.3 KW, respectively. The values (g Trolox/100 g DW) were 7.37 ± 0.17, 9.13 ± 0.11, 7.96 ± 0.07, 5.88 ± 0.30, and 5.83 ± 0.37, at 2.0 kW (after 10.0 min), 1.5 kW (35.0 min), 1.0 kW (50.0 min), 0.5 kW (100.0 min), and 0.3 kW (180.0 min), respectively. The highest FRAP value was observed at the same time point as the highest TPC. Further, FRAP values were significantly correlated with TPC (*R*^2^ = 0.916, *p* < 0.01) but not with ABTS (*R*^2^ = 0.249, *p* < 0.01). The ABTS value of green coffee beans was 9.97 ± 3.57 g Trolox/100 g DW. The values of green coffee beans in terms of Trolox equivalents were higher than those obtained using the DPPH and FRAP assays. The ABTS value remained nearly constant during microwave drying at 0.3 kW, 0.5 kW, and 1.0 kW. However, it tended to increase first and then decrease as the drying progressed during MVD at power settings of 1.5 kW and 2.0 kW (Figure 5d). The highest ABTS values were observed after 25.0 min at 1.5 kW (12.56 ± 1.56 g Trolox/100 g DW) and after 15.0 min at 2.0 kW (20.62 ± 0.06 g Trolox/100 g DW). The ABTS values showed little correlation with TPC, DPPH, and FRAP.

## 3. Materials and Methods

### 3.1. Materials

Coffee cherries (*Coffea* *canephora*) were harvested in March 2017 at the experimental station in the Spice and Beverage Research Institute of the Chinese Academy of Tropical Agricultural Sciences (Hainan, China). All samples were fresh, full, healthy, and red. Green coffee beans were obtained after peel removal and mucilage of the coffee cherry, and were stored at −20 °C. The average initial moisture content of the green coffee beans was 1.18 ± 0.01 g/g dry basis (db), as determined by the fast moisture analyzer (MB25; OHAUS, Shanghai, China).

### 3.2. MVD

A multifunctional and stable microwave vacuum dryer (Type WBZ-10PLC) purchased from Guizhou Xinqi Microwave Industry Co., Ltd. (Guiyang, Guizhou, China) was used in the present study. The loss of water content per h was 1.60, 1.20, 0.80, 0.35, and 0.20 kg/h at microwave power of 2.0 kW, 1.5 kW, 1.0 kW, 0.5 kW, and 0.3 kW, respectively. Samples (2 kg) were spread uniformly in a single thin layer on two plates, and the vacuum pump was turned on until the pressure reached −0.085 mPa. Five different microwave power settings (0.3 kW, 0.5 kW, 1.0 kW, 1.5 kW, and 2.0 kW) were used to investigate the drying characteristics of green coffee beans. The drying process was continued until the moisture content of green coffee beans fell below 10.0–11.0% (g/100 g dry weight (DW)). Next, the microwave vacuum-dried green coffee beans were ground with a micro-hammer mill, sieved through a 40-mesh screen, and stored in sealed kraft bags at 4 °C until further analysis.

### 3.3. Mathematical Modeling

The moisture ratio (MR) of dried samples at any time was calculated according to the following equation:

(1) $$MR=\frac{\left(M_{t}-M_{e}\right)}{\left(M_{0}-M_{e}\right)}$$

where *M_t_* is the moisture content at any time (kg water·kg dry matter^−1^); *M*_0_ is initial moisture content (kg water·kg dry matter^−1^); and *M_e_* is the equilibrium moisture content (kg water·kg dry matter^−1^).

The drying rate (DR) of green coffee beans at any time was calculated according to the following equation:

(2) $$DR=\frac{\left(W_{t}-W_{i}\right)}{\left(t-i\right)}$$

where *t*, and *i* are the drying time, *i*; *W_t_* is the moisture content at time *t* (kg water·kg dry matter^−1^); and *W_i_* is the moisture content at time *i* (kg water·kg dry matter^−1^).

To model the experimental data, 10 thin-layer drying models were chosen, as detailed in Supplemental Table S4. Regression analysis was performed using SPSS 20.0 (IBM SPSS Statistics for Windows, Version 20.0., IBM Corp., Armonk, NY, USA). The ability of each model to simulate the experimental data was evaluated using the correlation coefficient (*R*^2^), reduced chi-square (χ^2^), and root mean square error (RMSE). The best model was the one with the highest *R*^2^ value, and the lowest χ^2^ and RMSE values.

### 3.4. The Effective Moisture Diffusion Coefficient D_eff_

The effective moisture diffusion coefficient, an important, generally accepted kinetic parameter, describes the transport of moisture from the material to its surroundings, and can be calculated using the following equation:

(3) $$MR=\frac{8}{\pi }exp\left(-\frac{D_{eff}\cdot t\cdot \pi^{2}}{4L^{2}}\right)$$

which can be written as:

(4) $$D_{eff}=L^{2}\times \left(-0.101lnMR-0.0213\right)/t$$

where *D_eff_* is the effective moisture diffusion coefficient (cm^2^/min); *MR* represents the moisture content; *L* is the sample thickness (cm); and *t* is the drying time (min).

### 3.5. Surface Temperature

Surface temperature of the beans was determined using a non-contact infrared thermometer (8895, Hengxin Technology Co., Ltd., yixing, China). The surface temperature of each sample was determined at the end of each drying period. Establishment of the surface temperature kinetics greatly facilitates the study of temperature processing and chemical properties of green coffee beans during MVD. In the present study, the Allometric 1 model was employed to investigate changes in the surface temperature of green coffee beans during MVD. It can be expressed by the following equation:

(5) $$T=k\cdot t^{b}$$

where *T* is the surface temperature of the material (°C); *t* is the drying time (min); and *k* and *b* are constants.

### 3.6. Color

The color of green coffee beans and coffee powder dried under different drying conditions were determined using a colorimeter (X-Rite, SP62, Grand Rapids, Michigan, USA) based on the CIELAB method. Δ*E*, total color differences, was determined using the following equation:

(6) ΔE=(Lt−L∗)2+(at−a∗)2+(bt−b∗)2

where L* represents the lightness of the luminance component; a* and b* parameters (red to green, and yellow to blue, respectively) represent the two chromatic components; and *t* refers to the time point of color reading of green coffee beans, used as a control. The larger Δ*E*, the greater the color change from the reference color. All measurements were performed in triplicate.

### 3.7. Infrared Thermal Imaging

Infrared thermal imaging is an advanced non-destructive testing method, which is applied in the field of food processing. In the present study, infrared thermal imaging was used to analyze the temperature distribution in coffee samples during drying. A thermal imaging (SKF TKTI 21, Meike Technology Co., Ltd., Hongkong, China) was used in these determinations.

### 3.8. Differential Scanning Calorimetry

Thermal properties of the coffee bean samples were determined by DSC (DSC1, Mettler Toledo, Switzerland). In brief, samples (3.0 mg) from each treatment were encapsulated in aluminum DSC pans, following which the pans were hermetically sealed and equilibrated for 1 h at room temperature (25.0 ± 2.0 °C) The pans were then placed on a sample plate and heated from 20 to 250 °C, at 10 °C/min. Empty aluminum pan was used as a reference [29]. All measurements were performed in triplicate.

### 3.9. NMR

NMR relaxation measurements were performed as described by Zhao et al. [5], using a low-field pulsed NMI 20-Analyst (PQ001-20-025, Niumag Electric Corporation, Shanghai, China). Carr-Purcell-Meiboom-Gill sequences in an ^1^H NMR spectrometer were employed to measure the spin–spin relaxation time, T2. The transverse relaxation spectra were obtained from the Carr-Purcell-Meiboom-Gill decay curves reflecting the echo intensity. NMR imaging was also used to analyze water distribution using an NMR imaging analyzer (NMI20-015V-I, Niumag Electric Corporation).

### 3.10. Scanning Electron Microscopy

The microstructure of sample powder treated at different microwave power settings was characterized by SEM (SU1510, Hitachi, Tokyo, Japan). The dried coffee powder was placed on a metal stub and coated with gold powder to render the sample conductive. The surface of the prepared samples was visualized using SEM.

### 3.11. Analysis of the TPC and Antioxidant Activity

#### 3.11.1. Extraction of Phenolic Compounds

Dried coffee beans (0.5 g) from different drying stages were incubated for 3 h at 80 °C in 30.0 mL of 80% ethanol. TPC, 2,2′-diphenyl-1-picrylhydrazyl (DPPH) free radical-scavenging activity, ferric-reducing antioxidant power (FRAP), and 2,2′-azinobis-(3-ethyl-benzothiazoline-6-sulfonic acid) (ABTS) antioxidant activity were analyzed in the extracts as described below.

#### 3.11.2. TPC Determination

TPC of green coffee beans was quantified using the Folin-Ciocalteu colorimetric method [30], with minor modifications. In brief, 1.0 mL of sample extracts were transferred into a series of 25.0 mL colorimetric tubes containing 5.0 mL of 10.0% Folin-Ciocalteu reagent. After 4 min, the following were added to the mixtures: 4.0 mL of 7.5% Na_2_CO_3_ and 15.0 mL of deionized water, and the reaction was allowed to proceed for 90 min at room temperature. Sample absorbance was determined at 750 nm using a UV-Vis spectrophotometer (SPECORD 250 PLUS, Analytik Jena, Jena, Germany). Methanol (80%) was used as a blank. TPC was expressed as gallic acid equivalents (g GAE/100 g DW).

#### 3.11.3. Determination of DPPH Radical-Scavenging Activity

The DPPH assay determines the ability of antioxidants to scavenge the stable radical DPPH. The assay was conducted as described by Dong et al. [31], with some modifications. The sample extract was diluted five-fold with 80% ethanol; then, 0.1 mL of the diluted extract was added to 0.5 mL of a 0.6 mM solution of DPPH in methanol, and shaken vigorously. The mixture was combined with 4.4 mL of 80% methanol and incubated in the dark for 30 min. Sample absorbance was determined at 515 nm and the DPPH assay data expressed as g Trolox/100 g DW.

#### 3.11.4. FRAP Determination

The FRAP assay was performed as reported by Mahzir et al. [32], with small modifications. The stock solution contained 0.3 M sodium acetate buffer (pH 3.6), 10 mM 2,4,6-tripyridyl-s-triazine in 40 mM HCl, and 20 mM FeCl_3_ in distilled water, mixed at a ratio 10:1:1. Sample extract (0.1 mL) and ethanol (0.9 mL) were mixed with 4.0 mL of the stock solution, and the reaction allowed to proceed for 6 min at room temperature. FRAP was determined by monitoring the absorbance at 593 nm, with the FRAP assay data expressed as g Trolox/100 g DW.

#### 3.11.5. Determination of the ABTS Antioxidant Activity

The ABTS antioxidant activity was determined using the method of Zheng et al. [27], with some modifications. ABTS stock solution was prepared by dissolving 19.2 mg of ABTS in 5 mL of 2.45 mM potassium persulfate; the mixture was incubated in the dark for 12–16 h to generate the ABTS^+^ working solution. This solution was further diluted with 80% methanol for an absorbance of 0.70 ± 0.02 at 734 nm. Sample extract (0.1 mL) and 80% methanol (0.9 mL) were mixed with 4 mL of the diluted ABTS^+^ solution, and allowed to react for 10 min at room temperature. ABTS absorbance was determined at 734 nm, with the ABTS assay data expressed as g Trolox/100 g DW.

### 3.12. Statistical Analysis

All experimental data were analyzed using Origin 8.0 (Microcal Software, Inc., Northampton, MA, USA) and SPSS 20.0 (Chicago, IL, USA). Significant differences between samples were determined using the analysis of variance (ANOVA) and Duncan’s multiple-range test (*p* < 0.05). Each sample was analyzed in triplicate, the mean values were used in statistical analysis, and expressed as the mean ± standard deviation (SD).

## 4. Conclusions

In the present study, the effect of microwave power on the drying characteristics of green coffee beans was investigated. The drying time was reduced about 7.8-fold when the microwave power was decreased from 2.0 kW to 0.3 kW. The best fitting model for kinetics data for green coffee beans submitted to MVD was the Page model. Moreover, high microwave power during MVD (2.0 kW, 1.5 kW, and 1.0 kW) resulted in slow-fast-slow water removal during drying; in contrast, low microwave power during MVD (0.5 and 0.3 kW) resulted in an almost uniform speed of water removal during drying. The surface temperature rise kinetics can provide the link between temperature and the bioactive component content, and require further study. Infrared thermal imaging of coffee beans dried at 1.0 kW revealed heterogeneity of surface temperature that became apparent after 60.0–75.0 min of drying. NMR and MRI analyses revealed that the water content and distribution state changed during MVD, with the greatest changes concerning cytoplasmic bulk water and bound water. Thermal processing of green coffee beans resulted in increased b*, L*, Δ*E*, and TPC values, as well as increased antioxidant capacity. On the other hand, the a* value remained largely unchanged during MVD. As demonstrated in the present study, TPC and antioxidant activity was higher in, and the color significantly different for, coffee beans dried using MVD at higher microwave power than beans treated at lower power.

## Figures and Tables

**Table 1:** Equation (5) coefficient and the effective moisture diffusion coefficient.

Microwave Power (kW)	a	b	*R* ^2^	RMSE	*χ* ^2^	*D_eff_* (cm^2^/min)
0.3	21.41	0.17	0.88	31.12	4.45	1.13 × 10^−3^
0.5	25.22	0.18	0.88	35.63	5.94	1.70 × 10^−3^
1.0	35.78	0.15	0.97	7.73	1.29	3.78 × 10^−3^
1.5	40.57	0.14	0.98	6.39	0.80	5.57 × 10^−3^
2.0	39.51	0.15	0.97	51.45	10.29	6.62 × 10^−3^

## Supplementary Materials

The following are available online, Figure S1: DSC spectra of coffee beans dried at five different microwave power settings. Figure S2: NMR imaging of samples at six stages of the drying process at 1.0-kW microwave power: (a) 0 min; (b) 15.0 min; (c) 30.0 min; (d) 45.0 min; (e) 60.0 min; and (f) 75.0 min. Figure S3: Infrared thermal imaging of samples at eight stages of the drying process at 1.0 kW microwave power: (a) 0 min; (b) 10.0 min; (c) 20.0 min; (d) 30.0 min; (e) 40.0 min; (f) 50.0 min; (g) 60.0 min; and (h) 75.0 min. Table S1: Statistical analyses of modelling of the moisture ratio and drying time during MVD. Table S2: Peak area ratios for the three states of water. Table S3: The Person correlation coefficients for different chemical properties of microwave-dried coffee beans. Table S4: Ten thin-layer drying models used for experimental data fitting [33,34,35,36,37,38].
