# Supplementary material for: Effect of Microwave Vacuum Drying on the Drying Characteristics, Color, Microstructure, and Antioxidant Activity of Green Coffee Beans
Source: Molecules. 2018 May 11;23(5):1146. doi: 10.3390/molecules23051146 (PMC6100066; doi:10.3390/molecules23051146)

**Supplementary material**

**Supplemental Table 1.** Ten thin-layer drying models used for experimental data fitting.

| NO. | Model name | Model | Reference |
|---|---|---|---|
| 1 | Page | $MR=Exp(-kt^n)$ | Rodriguez, & Cleland, 1988 [33] |
| 2 | Lewis | $MR=Exp(-kt)$ | Midilli, Kucuk, & Yapar, 2002 [34] |
| 3 | Henderson &Pabis | $MR=aExp(-kt)$ | Xanthopoulos et al., 2007 [35] |
| 4 | Modified page | $MR=Exp(-kt)^n$ | Midilli, Kucuk, & Yapar, 2002 [34] |
| 5 | Wang & Singh | $MR=1+at+bt^2$ | Midilli, Kucuk, & Yapar, 2002 [34] |
| 6 | Approximation of diffusion | $MR=aExp(-kt)+(1-a) Exp(-kat)$ | Contini, Martelli, & Zaccanti, 1997 [36] |
| 7 | Logarithmic | $MR= aExp(-kt)+c$ | Midilli, Kucuk, & Yapar, 2002 [34] |
| 8 | Simplified Fick's diffusion | $MR=aExp(-c(t/l^2))$ | Midilli, Kucuk, & Yapar, 2002 [34] |
| 9 | Modified Page equation-II | $MR=Exp(-c(t/l^2)^n)$ | Wang et al.,2007 [37] |
| 10 | Two-term model | $MR=aExp(-k_0t)+ bExp(-k_1t)$ | Mohapatra, & Rao, 2005 [38] |

**Supplemental Table 2.** Statistical analyses of modelling of the moisture ratio and drying time during MVD.

| Drying condition | | Page | Lewis | Modified page | Wang & Singh | Logarithmic | Henderson & Pabis | Approximation of diffusion | Simplified Fick's diffusion | Modified Page equation-II | Two-term Model |
|---|---|---|---|---|---|---|---|---|---|---|---|
| 0.3 kW | $R^2$ | 0.9930 | 0.9271 | 0.9180 | 0.9866 | **0.9950** | 0.9563 | 0.9180 | 0.9501 | 0.9920 | 0.9418 |
| | *RMSE* | 0.0057 | 0.0672 | 0.0672 | 0.0110 | **0.0036** | 0.0358 | 0.0672 | 0.0358 | 0.0057 | 0.0358 |
| | $\chi^2$ | 7.16E-4 | 0.0075 | 0.0084 | 0.0014 | **5.14E-4** | 0.0045 | 0.0084 | 0.0051 | 8.17E-4 | 0.0060 |
| 0.5 kW | $R^2$ | **0.9912** | 0.9367 | 0.9276 | 0.9799 | 0.9872 | 0.9615 | 0.9276 | 0.9551 | 0.9897 | 0.9461 |
| | *RMSE* | **0.0055** | 0.0450 | 0.0450 | 0.0125 | 0.0068 | 0.0239 | 0.0450 | 0.0239 | 0.0055 | 0.0239 |
| | $\chi^2$ | **7.82E-4** | 0.0056 | 0.0064 | 0.0018 | 0.0011 | 0.0034 | 0.0064 | 0.0040 | 9.12E-4 | 0.0048 |
| 1.0 kW | $R^2$ | **0.9927** | 0.9303 | 0.9203 | 0.9772 | 0.9858 | 0.9573 | 0.9203 | 0.9502 | 0.9915 | 0.9402 |
| | *RMSE* | **0.0050** | 0.0552 | 0.0552 | 0.0158 | 0.0084 | 0.0296 | 0.0553 | 0.0296 | 0.0050 | 0.0296 |
| | $\chi^2$ | **7.19E-4** | 0.0069 | 0.0079 | 0.0023 | 0.0014 | 0.0042 | 0.0079 | 0.0049 | 8.39E-4 | 0.0059 |
| 1.5 kW | $R^2$ | 0.9944 | 0.9486 | 0.9428 | 0.9816 | 0.9916 | 0.9760 | **0.9956** | 0.9730 | 0.9937 | 0.9691 |
| | *RMSE* | 0.0041 | 0.0426 | 0.0426 | 0.0137 | 0.0056 | 0.0179 | **0.0033** | 0.0179 | 0.0041 | 0.0179 |
| | $\chi^2$ | 4.60E-4 | 0.0043 | 0.0047 | 0.0015 | 6.94E-4 | 0.0020 | **3.65E-4** | 0.0022 | 5.18E-4 | 0.0026 |
| 2.0 kW | $R^2$ | **0.9928** | 0.9179 | 0.9042 | 0.9738 | 0.9873 | 0.9548 | 0.7588 | 0.9458 | 0.9913 | 0.9323 |
| | *RMSE* | **0.0036** | 0.0478 | 0.0478 | 0.0131 | 0.0053 | 0.0226 | 0.1204 | 0.0226 | 0.0036 | 0.0226 |
| | $\chi^2$ | **6.01E-4** | 0.0068 | 0.0080 | 0.0022 | 0.0011 | 0.0038 | 0.0201 | 0.0045 | 7.21E-4 | 0.0056 |

Undertake the above Table.

**Supplemental Table 3.** Peak area ratios for the three states of water.

| Microwave power (kW) | Drying time (min) | $A_{21}$ (%) | $A_{22}$ (%) | $A_{23}$ (%) |
|---|---|---|---|---|
| 0.3 kW | 0 | 0.210 | 0.698 | 0.092 |
| | 56.0 | 0.251 | 0.702 | 0.047 |
| | 112.0 | 0.974 | 0.000 | 0.026 |
| | 168.0 | 0.930 | 0.000 | 0.070 |
| | 224.0 | 0.760 | 0.113 | 0.127 |
| | 280.0 | 0.655 | 0.340 | 0.005 |
| 0.5 kW | 32.0 | 0.000 | 0.935 | 0.065 |
| | 64.0 | 0.933 | 0.000 | 0.067 |
| | 96.0 | 0.813 | 0.123 | 0.063 |
| | 128.0 | 0.805 | 0.115 | 0.080 |
| | 160.0 | 0.826 | 0.000 | 0.174 |
| 1.0 kW | 15.0 | 0.000 | 0.938 | 0.062 |
| | 30.0 | 0.978 | 0.000 | 0.022 |
| | 45.0 | 0.845 | 0.093 | 0.062 |
| | 60.0 | 0.832 | 0.116 | 0.052 |
| | 75.0 | 0.776 | 0.125 | 0.100 |
| 1.5 kW | 10.0 | 0.000 | 0.907 | 0.093 |
| | 20.0 | 0.956 | 0.000 | 0.044 |
| | 30.0 | 0.871 | 0.110 | 0.019 |
| | 40.0 | 0.833 | 0.129 | 0.038 |
| | 50.0 | 0.709 | 0.237 | 0.054 |
| 2.0 kW | 7.0 | 0.017 | 0.925 | 0.058 |
| | 14.0 | 0.974 | 0.000 | 0.026 |
| | 21.0 | 0.866 | 0.093 | 0.041 |
| | 28.0 | 0.855 | 0.084 | 0.060 |
| | 36.0 | 0.797 | 0.102 | 0.101 |

**Supplemental Table 4.** The Person correlation coefficients for different chemical properties of microwave-dried coffee beans.

| | TPC | DPPH | FRAP | ABTS |
|---|---|---|---|---|
| TPC | 1.000 | | | |
| DPPH | 0.660** | 1.000 | | |
| FRAP | 0.916** | 0.706** | 1.000 | |
| ABTS | 0.180 | 0.070 | 0.249 | 1.000 |

**$p < 0.01$.

**Supplemental Figure 1.** DSC spectra of coffee beans dried at five different microwave power settings.

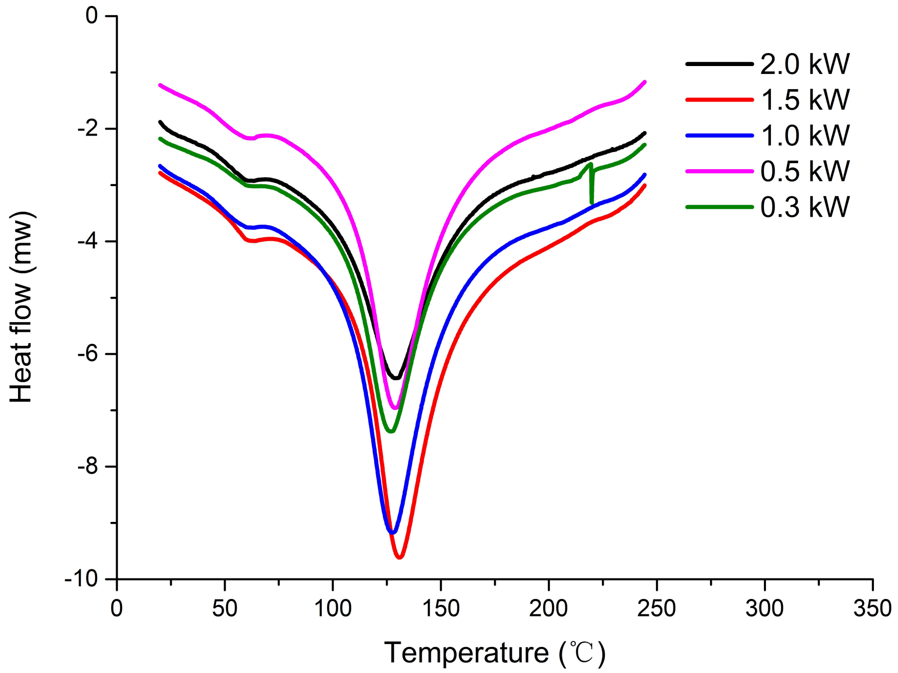

**Supplemental Figure 2.** NMR imaging of samples at six stages of the drying process at 1.0-kW microwave power: (a) 0 min; (b) 15.0 min; (c) 30.0 min; (d) 45.0 min; (e) 60.0 min; and (f) 75.0 min.

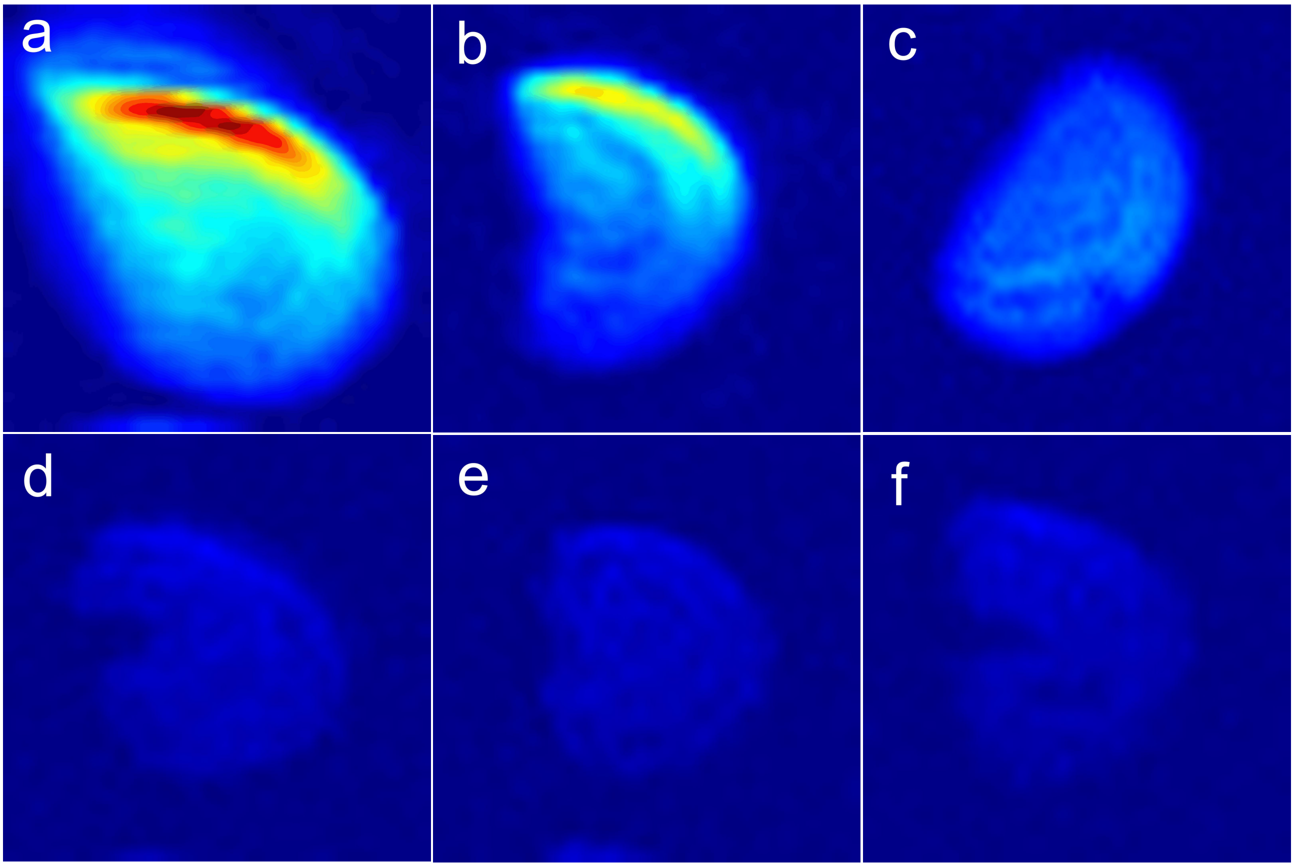

**Supplemental Figure 3.** Infrared thermal imaging of samples at eight stages of the drying process at 1.0 kW microwave power: (a) 0 min; (b) 10.0 min; (c) 20.0 min; (d) 30.0 min; (e) 40.0 min; (f) 50.0 min; (g) 60.0 min; and (h) 75.0 min.

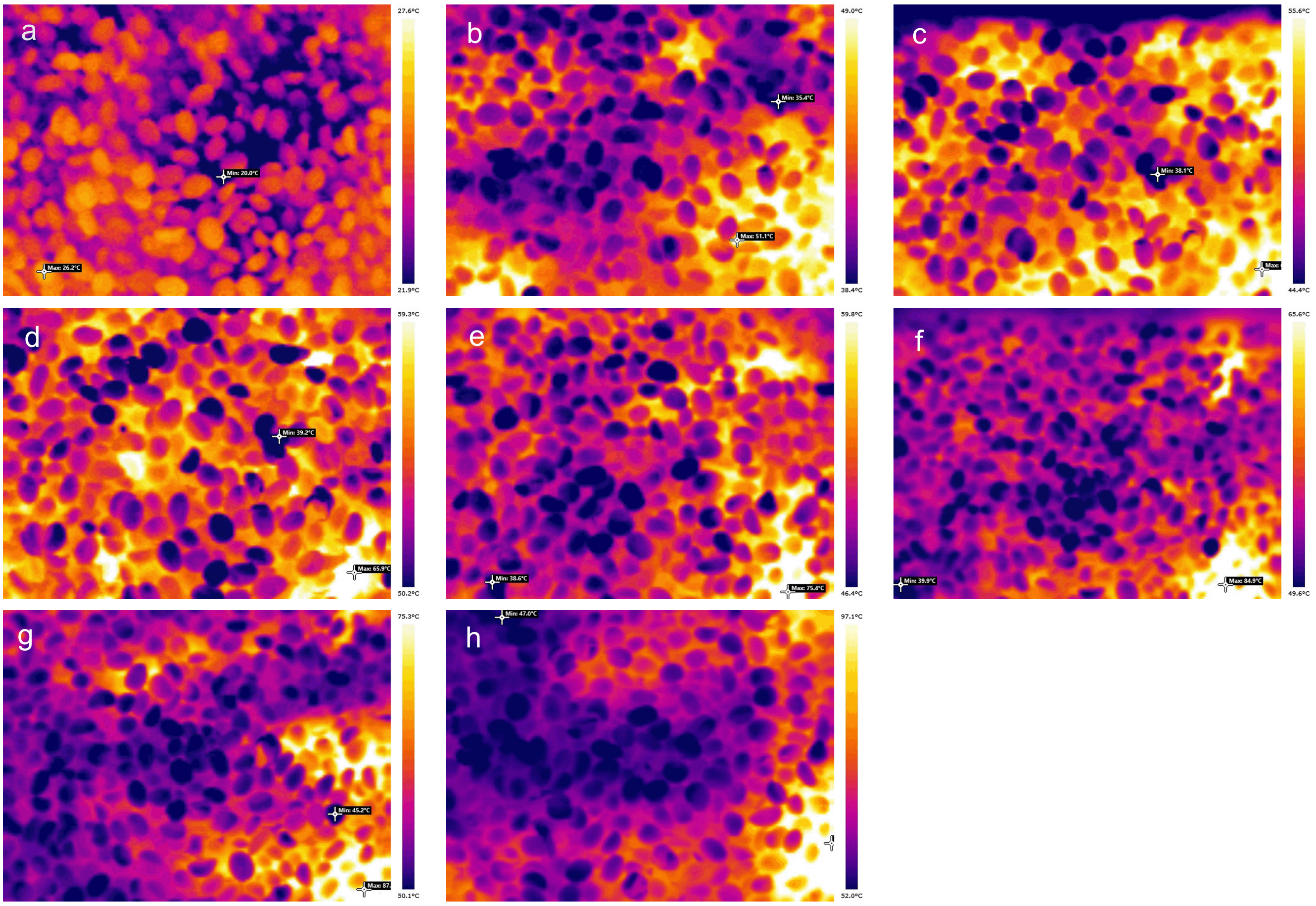

Supplement: Supplementary file 1 [file molecules-23-01146-s001.pdf]
